# Regenerative therapies in rehabilitation with interfaces with occupational medicine, geriatrics, and oncology: the example of extracorporeal shock wave therapy

**DOI:** 10.1007/s10354-026-01169-0

**Published:** 2026-06-23

**Authors:** Richard Crevenna

**Affiliations:** 1 Department of Physical Medicine, Rehabilitation and Occupational Medicine, Medical University of Vienna, University Hospital General Hospital Vienna, Vienna Healthcare Group, Vienna, Austria; 2 Comprehensive Center for Musculoskeletal Disorders (CCMSD), Medical University of Vienna, University Hospital General Hospital Vienna, Vienna Healthcare Group, Vienna, Austria; 3 Competence Center for Occupational Safety and Health Promotion, University Hospital General Hospital Vienna, Vienna Healthcare Group, Vienna, Austria

**Keywords:** Extracorporeal shockwave therapy, Regeneration, Rehabilitation, Occupational medicine, Geriatrics, Extrakorporale Stoßwellentherapie, Regeneration, Rehabilitation, Arbeitsmedizin, Geriatrie

## Abstract

**Background:**

Regenerative therapies are increasingly integrated into modern rehabilitation medicine. Extracorporeal shock wave therapy (ESWT) represents a central modality within this framework, extending beyond symptom control toward enabling functional recovery across disciplines including occupational medicine, geriatrics, and oncology. In addition, its potential socioeconomic impact is of growing relevance.

**Methods:**

This work comprises a narrative synthesis integrating concepts of regenerative medicine, rehabilitation, prehabilitation, prevention, and health economics, with a focus on ESWT as a mechanotherapeutic intervention.

**Results:**

Rehabilitation is based on a biopsychosocial model targeting body functions, activities, and participation. By reducing pain, modulating inflammation, and promoting tissue regeneration, ESWT acts as a biological “enabler” thereby, facilitating active rehabilitation strategies. In oncological settings, ESWT may support the management of treatment-related functional impairments. In occupational medicine and geriatrics, it contributes to maintaining ability to work and independence. From a socioeconomic perspective, ESWT may reduce healthcare utilization, prevent chronicity, shorten the duration of rehabilitation, and facilitate return to work, thereby lowering indirect costs. While evidence for primary prevention is lacking, ESWT is relevant in tertiary and quaternary prevention.

**Conclusion:**

Extracorporeal shock wave therapy represents a key component of regenerative rehabilitation strategies. Its function-oriented and potentially cost-effective application enhances mobility, participation, and quality of life, particularly in ageing and multimorbid populations.

## Abstract (de)

**Hintergrund:**

Regenerative Therapien werden zunehmend in die moderne Rehabilitationsmedizin integriert. Die extrakorporale Stoßwellentherapie (ESWT) stellte eine zentrale Modalität in diesem Rahmen dar, sie erstreckt sich über die reine Symptomkontrolle hinaus auf die Ermöglichung der funktionellen Genesung über verschiedene Fachgebiete hinweg einschließlich Arbeitsmedizin, Geriatrie und Onkologie. Außerdem ist ihr Potenzial für sozioökonomische Auswirkungen von zunehmender Relevanz.

**Methoden:**

Die vorliegende Arbeit umfasst eine narrative Synthese integrativer Konzepte der regenerativen Medizin, Rehabilitation, Prähabilitation, Prävention und Gesundheitsökonomie; der Fokus liegt dabei auf der ESWT als einer mechanotherapeutischen Intervention.

**Ergebnisse:**

Rehabilitation basiert auf einem biopsychosozialen Modell, das auf Körperfunktionen, Aktivitäten und Teilhabe abzielt. Durch Schmerzlinderung, Beeinflussung von Entzündungsprozessen und Förderung der Geweberegeneration wirkt die ESWT wie ein biologischer „Wegbereiter“, der so aktive Rehabilitationsstrategien erleichtert. In onkologischen Zusammenhängen kann die ESWT die Behandlung therapiebedingter funktioneller Beeinträchtigungen unterstützen. In der Arbeitsmedizin und der Geriatrie trägt sie zur Erhaltung der Arbeitsfähigkeit und Unabhängigkeit bei. Aus sozioökonomischer Sicht kann die ESWT die Inanspruchnahme des Gesundheitswesens vermindern, einer Chronifizierung vorbeugen, die Dauer der Rehabilitation verkürzen und die Rückkehr ins Berufsleben erleichtern und somit die indirekten Kosten senken. Es fehlt zwar noch Evidenz für die Primärprävention, aber in der Tertiär- und Quartärprävention ist die ESWT relevant.

**Schlussfolgerung:**

Die ESWT stellt eine Schlüsselkomponente der regenerativen Rehabilitationsstrategien dar. Ihre funktionsorientierte und potenziell kostengünstige Anwendung fördert die Mobilität, Teilhabe und Lebensqualität, insbesondere in alternden und multimorbiden Populationen.

## Key messages

- Extracorporeal shock wave therapy (ESWT) represents a regenerative and function-enabling therapy within multimodal rehabilitation concepts.
- Pain reduction through ESWT as an “enabler” facilitates active therapy and functional recovery, and rehabilitation should be understood within a biopsychosocial framework focusing on participation.
- Extracorporeal shock wave therapy has particular relevance in oncology, occupational medicine, and geriatrics.
- Use of ESWT may have substantial socioeconomic benefits.

## Introduction

Regenerative therapies are reshaping rehabilitation medicine by shifting the focus from structural pathology toward functional recovery and participation. The concept of “mobility equals participation” emphasizes that mobility constitutes a prerequisite for social inclusion, the ability to work, and quality of life.

Extracorporeal shock wave therapy (ESWT), as a modality of mechanotherapy, exemplifies this paradigm. Its clinical relevance is increasing in parallel to demographic ageing, the rising prevalence of chronic musculoskeletal disorders, and growing socioeconomic pressure on healthcare systems [1, 2].

## Regenerative therapies and mechanotherapy

Regenerative therapies aim to activate endogenous repair mechanisms rather than replace tissue. Within physical medicine, mechanotherapy plays a central role by applying mechanical stimuli to induce biological responses. Extracorporeal shock wave therapy induces angiogenesis, modulates inflammatory processes, and promotes tissue regeneration through mechanotransduction. These biological effects are accompanied by clinically relevant analgesia, forming the basis for its functional application [1–12].

## Assessment and outcome measurement

Assessment should include clinical examination, pain evaluation, and validated functional outcome measures. In addition to clinical parameters, functional independence and return to work should be considered as key outcome indicators [1, 2].

## Practical implementation

The clinical use of ESWT requires appropriate indication, adherence to established guidelines, and precise application. Importantly, ESWT should be integrated into multimodal rehabilitation programs and combined with active therapeutic interventions [1, 2].

## Rehabilitation as a biopsychosocial and socioeconomic process

Rehabilitation encompasses medical, therapeutic, and social interventions aimed at restoring function and quality of life. According to the International Classification of Functioning, Disability and Health (ICF) model, rehabilitation operates across the domains of body functions and structures, activities, and participation. Participation includes social integration, independence, and economic productivity. Consequently, rehabilitation inherently possesses a socioeconomic dimension. Loss of mobility and function is associated with reduced ability to work, increased healthcare utilization, and a higher risk of long-term disability and care dependency. Effective rehabilitation strategies must therefore address both clinical and socioeconomic outcomes.

## Extracorporeal shock wave therapy as a therapeutic enabler

Extracorporeal shock wave therapy functions as a biological “enabler” by reducing pain and improving tissue conditions, thereby allowing patients to engage in active rehabilitation and exercise. Pain reduction leads to improved mobility, which in turn facilitates participation in structured exercise programs. This enables progressive functional recovery and ultimately supports participation in daily life, work, and social activities. This concept is central to understanding both the clinical effectiveness and the broader socioeconomic relevance of ESWT [1–12; Table 1].

**Table 1:** Extracorporeal shock wave therapy (ESWT) as an “enabler” in functional rehabilitation pathways

Step	Clinical process	Role of ESWT	Outcome
1	Pain-limited condition	Pain modulation	Improved tolerance to movement
2	Initiation of therapy	Facilitates active rehabilitation	Increased participation in training
3	Functional progression	Supports load adaptation	Improved strength and endurance
4	Reintegration	Enables return to work/participation	Sustainable functional recovery

## Extracorporeal shock wave therapy in musculoskeletal rehabilitation

In musculoskeletal rehabilitation, ESWT contributes to pain reduction, functional restoration, and the prevention of chronic conditions. Typical indications include tendinopathies, plantar fasciitis, chronic low back pain, and impaired bone healing. By enabling early mobilization and facilitating active therapy, ESWT may shorten the duration of rehabilitation and improve long-term functional outcomes [3–12].

## Extracorporeal shock wave therapy in oncological rehabilitation

Oncological rehabilitation is gaining importance due to increasing survival rates and the growing number of patients with long-term treatment-related impairments. Common sequelae include fibrosis, myofascial pain, reduced mobility, and decreased physical performance. Within this context, ESWT may contribute to improving tissue elasticity, reducing pain, and enabling participation in exercise-based rehabilitation programs. These effects are particularly relevant for addressing functional decline and cancer-related fatigue. Extracorporeal shock wave therapy is not applied in areas of active malignancy. However, in appropriate clinical settings, it may support functional recovery, improve quality of life, and enhance participation in cancer survivors (Table 2**)**.

**Table 2:** Extracorporeal shock wave therapy (ESWT) in rehabilitation (including oncological rehabilitation)

Domain	Clinical condition	Therapeutic target	Effect of ESWT	Clinical relevance
Musculoskeletal rehabilitation	Tendinopathies, plantar fasciitis, low back pain	Pain reduction, tissue regeneration	Improves load tolerance and function	Enables early mobilization and active therapy
Postoperative rehabilitation	After orthopedic surgery	Delayed recovery, pain	Facilitates functional recovery	Supports return to activity and participation
Oncological rehabilitation	Fibrosis, myofascial pain, mobility restriction	Pain, reduced mobility, fatigue	Improves tissue elasticity and reduces pain	Enables exercise therapy and improves participation
Supportive care oncology	Treatment-related functional impairment	Reduced physical performance	Indirect effect via enabling activity	Contributes to improved quality of life
General rehabilitation concept	Multimodal rehabilitation	Functional restoration	ESWT acts as “enabler” of active therapy	Must be combined with exercise and training

## Extracorporeal shock wave therapy in prehabilitation

Prehabilitation aims to improve patients’ functional capacity prior to planned medical interventions. The principle “better in results in better out” highlights that pre-interventional fitness is closely associated with postoperative outcomes. When pain limits physical training, ESWT may reduce symptoms sufficiently to allow participation in structured prehabilitation programs. This can enhance physical performance and improve postoperative recovery [1, 13; Table 3].

**Table 3:** Extracorporeal shock wave therapy (ESWT) in prehabilitation (including oncological settings)

Clinical context	Typical indication	Functional limitation	Role of ESWT	Integration into prehabilitation
Orthopedic prehabilitation	Tendinopathies before joint replacement	Pain-limited mobility and training capacity	Pain reduction enabling exercise training	Combined with strength, endurance, and balance training
Oncological prehabilitation	Musculoskeletal pain before cancer treatment or surgery	Reduced physical capacity, fatigue, movement restriction	Enables participation in structured exercise programs	Integrated with multimodal prehabilitation (nutrition, psycho-oncology, exercise)
Multimorbid patients	Chronic musculoskeletal pain before interventions	Low baseline functional capacity	Facilitates training tolerance and adherence	Individualized, interdisciplinary approach
General principle	“Better in results in better out”	Limited pre-interventional fitness	ESWT as an enabling tool rather than a primary intervention	Always combined with active training and rehabilitation concepts

## Extracorporeal shock wave therapy in prevention

There is currently no evidence supporting the use of ESWT in primary prevention. However, ESWT plays a relevant role in tertiary prevention by stabilizing function and reducing recurrence in chronic conditions. In addition, it contributes to quaternary prevention by reducing the need for invasive procedures and thereby helps to avoid overtreatment [2, 14, 15].

## Interfaces with occupational medicine

In occupational medicine, ESWT supports the maintenance and restoration of ability to work. By improving function and reducing pain-related limitations, it facilitates return to work and helps to prevent long-term disability, particularly in patients with chronic musculoskeletal disorders [1, 14, 15; Table 4].

**Table 4:** Extracorporeal shock wave therapy (ESWT) in occupational medicine

Occupational domain	Typical exposure	Clinical condition	Role of ESWT	Occupational outcome
Healthcare workers	Lifting, repetitive strain	Shoulder and back disorders	Reduces pain and improves function	Facilitates return to work and workload tolerance
Manual labor (construction, industry)	Vibration, overload, repetitive work	Tendinopathies, enthesopathies	Supports tissue regeneration and pain control	Reduces absenteeism and chronicity
Office work	Sedentary posture, repetitive movements	Myofascial pain syndromes	Improves mobility and reduces pain	Enhances work performance
Work-related injuries	Trauma, delayed healing	Non-unions, chronic pain	Supports healing processes	Accelerates reintegration into the workforce
General role	Work disability risk	Functional limitation	ESWT as adjunct in rehabilitation	Improves return-to-work rates and reduces indirect costs

## Interfaces with geriatrics

In geriatric populations, mobility is a key determinant of independence and quality of life. Extracorporeal shock wave therapy contributes to improved mobility, a reduced fall risk, and maintenance of functional independence. Furthermore, it leads to better wound healing, especially in diabetic and multimorbid patients. These effects are highly relevant for reducing care dependency and optimizing resource utilization in ageing societies [16–19; Table 5].

**Table 5:** Extracorporeal shock wave therapy (ESWT) in geriatrics and care settings

Geriatric domain	Clinical problem	Therapeutic goal	Role of ESWT	Clinical and socioeconomic relevance
Mobility limitation	Pain-related immobility	Improve movement and independence	Reduces pain and facilitates mobilization	Reduces fall risk and improves quality of life
Degenerative conditions	Tendinopathies, osteoarthritis	Functional stabilization	Enhances tissue response and function	Supports long-term independence
Multimorbidity	Polypharmacy, reduced tolerance to medication	Noninvasive treatment option	Safe; low systemic burden	Reduces medication use and side effects
Frailty and sarcopenia (adjunct)	Reduced physical capacity	Support rehabilitation programs	Enables participation in exercise	Improves functional reserve
Care dependency	Reduced independence	Maintain autonomy	Indirect effect via improved mobility	Delays institutionalization and reduces care burden

## Socioeconomic relevance of extracorporeal shock wave therapy

The socioeconomic relevance of ESWT extends beyond individual patient outcomes. From a direct healthcare perspective, ESWT may reduce the need for surgical interventions, decrease medication use, and limit the utilization of diagnostic procedures. From an indirect perspective, it facilitates earlier return to work, reduces absenteeism and presenteeism, and lowers the risk of long-term disability. At the system level, maintaining mobility and independence contributes to prolonged workforce participation, reduced healthcare expenditure, and a decreased demand for long-term care. Thus, ESWT aligns with the principles of efficient and sustainable healthcare delivery [1].

## Conclusion

Regenerative therapies are transforming rehabilitation medicine by focusing on function, mobility, and participation. As a biological and functional enabler, ESWT exemplifies this approach. Extracorporeal shock wave therapy should not be understood as an isolated intervention, but rather as a therapeutic enabler within comprehensive preventive, treatment, and rehabilitation strategies. By facilitating active rehabilitation, it improves clinical outcomes while addressing broader socioeconomic challenges. In the context of demographic change and increasing chronic disease burden, ESWT represents a valuable component of sustainable, patient-centered healthcare.

## Data Availability

All data supporting the findings of this work are included in the article.
